# The Dual Role of USP11 in Cancer

**DOI:** 10.1155/2022/9963905

**Published:** 2022-03-22

**Authors:** Tuanjie Guo, Heting Tang, Zhihao Yuan, Encheng Zhang, Xiang Wang

**Affiliations:** Department of Urology, Shanghai General Hospital, Shanghai Jiao Tong University School of Medicine, Shanghai, China

## Abstract

Ubiquitination is one of the most crucial ways of protein degradation and plays an indispensable role in various living activities of cells. The deubiquitinating enzyme (DUB) is the main practitioner of the reversal of ubiquitination. Up till the present moment, nearly 100 DUBs from six families have been confirmed. USP11 is a member of the largest subfamily of cysteine protease DUBs, involving in the regulation of cell cycle, DNA repair, regulating signaling pathways, tumor development, and other important biological behaviors. This review briefly describes the structure and function of USP11 and comprehensively describes its dual role in tumorigenesis and development, as well as its targeted therapy.

## 1. Introduction

Ubiquitination can be divided into monoubiquitination and polyubiquitination, where polyubiquitination regulates protein stability, while monubiquitination is involved in protein localization, protein–protein interaction, and enzyme activity [1]. Protein degradation after ubiquitination is one of the most important protein degradation pathways in eukaryote. Ubiquitination and deubiquitination are reversible and mutually antagonizing processes, and their balance determines the homeostasis of proteins in cells. The degradation of proteins by ubiquitination requires the ubiquitin–proteasome system (UPS), which are protein complexes of ten to twenty subunits found in the cytoplasm and nucleus. The 26s proteasome is ubiquitous in eukaryotic cells. Its composition could be divided into two parts, one is the 20s proteasome, which is the core component, and the other is two 19s proteasomes, which play a regulatory role. Most of the proteins in the cell are degraded by the 26s proteasome. The ubiquitination and degradation of the target protein are completed through several consecutive processes. First, when ATP supplies energy, ubiquitin-activating enzyme E1 activates ubiquitin molecules. Then, ubiquitin-activating enzyme E1 transfers activated ubiquitin molecules to ubiquitin conjugating enzyme E2. After this, ubiquitin ligase E3 connects E2-bound ubiquitin to the target protein. In the end, the 26s proteasome specifically recognizes this substrate protein labeled with ubiquitin and degrades it [2, 3].

After protein is ubiquitinated, DUBs can reverse the process of ubiquitination by removing ubiquitin moieties from the targeted protein [4]. So far, scientists have found nearly 100 DUBs in humans, which are divided into six families. They are the ubiquitin-specific proteases (USPs), ubiquitin C-terminal hydrolases (UCHs), ovarian tumor proteases (OTUs), Machado–Joseph disease protein domain proteases (MJDs), JAMM/MPN domain-associated metallopeptidases (JAMMs) [4], and monocyte chemotactic protein-induced proteases (MCPIPs) [5]. Among the six families of DUBs, the USP family has the most members, and each of them has a variety of biological functions. In this review, we mainly describe the biological functions and behaviors of USP11, as well as its role in different cancers.

## 2. Structure, Subcellular Location, and Function of USP11

### 2.1. The Structure and Subcellular Location of USP11

**Figure 1:**
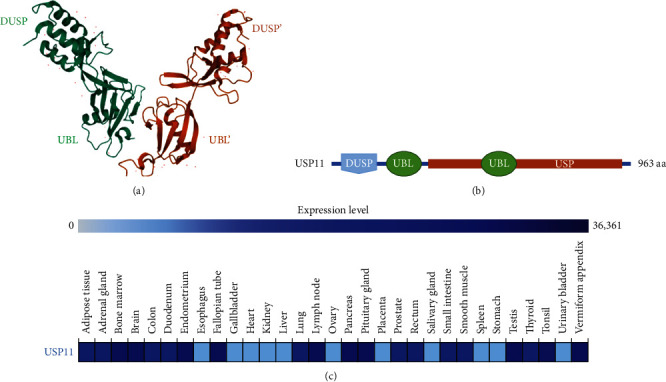
Structure of USP11 N-terminal DUSP-UBL domains and tissues distribution. (a) Three-dimensional structure of USP11 DUSP-UBL dimer. The second copy is labeled as ′. (b) Schematic representation of the domain composition for USP11. (c) USP11 has different expression levels in different tissues. Expression levels are indicated by color shades.

The earliest name of USP11 is UHX1. Swanson et al. identified its cDNA for the first time. They found that *UHX1* encodes an ubiquitin carboxyl-terminal hydrolase and is located on the Xp21.2-p11.2. The enzyme is highly expressed in retinal tissues and associated with X chromosome-linked retinal diseases [6]. This major discovery was verified by Brandau et al. [7]. There are 12 transcriptional versions of USP11, of which only four encode proteins, and their detailed information is presented in Table 1. As members of the DUSP-UBL (DU) family, USP11, USP4, and USP15 have the same two ubiquitin-like (UBL) domains and a N-terminal domain present in ubiquitin-specific proteases (DUSP) [8]. Unlike USP4 and USP15, the structure of USP11 has not been well studied until now. Compared with the homologues USP4 and USP15, the crystal structure of the DU domain of USP11 shows a tandem arrangement, with a shortened *β*-hairpin at the two-domain interface and different surface characteristics. Harper et al. found that the catalytic activity of USP11 is not regulated by intramolecular self-inhibition or activation by the N-terminal DU or UBL domain, and that the USP11 domain outside the catalytic core domain acts as a protein interaction or transport module without participating in direct regulation of proteolytic activity. In addition, a conserved VEVY motif is a hallmark feature on the two-domain interface, and its presence constitutes a potential protein interaction site [9]. This fully highlights the diversity of USPs in substrate recognition and ubiquitin dissociation regulation. Recently, researchers discovered that the crystal structure of the USP11 peptide complex revealed a previously unknown binding site in the noncatalytic ubiquitin-like (UBL) region of USP11. This site interacts with the helical motif, and the binding site regulates the function of USP11 in DNA repair mediated by homologous recombination [10]. The cartoon simulation diagram and three-dimensional structure of USP11 are shown in Figures 1(a) and 1(b) [9, 11].

USP11 is mainly located in the nucleus [12, 13]. When it interacts with some proteins to perform biological functions, it will be located on the chromatin [14, 15]. USP11 in the cytoplasm is only a negligible part [13]. Apart from this, USP11 has different expression levels in different tissues. The expression levels of USP11 in various tissues are shown in Figure 1(c) [16, 17].

### 2.2. The Function of USP11

#### 2.2.1. Proteins That Interact with USP11

USP11 exerts its biological function by contacting with target protein and interacting with it. RanBPM is the first protein discovered to interact with USP11, and they colocalize in the nucleus. RanBPM can be deubiquitinated by USP11, and it is also necessary for the correct nucleation of microtubules, which means that USP11 plays an irreplaceable role in cell division [12]. Subsequently, BRCA2, the second protein interacting with USP11, was discovered. The BRCA2 protein is believed to play an indispensable role in the repair of DNA double-strand break. Individuals carrying breast cancer susceptibility gene *BRCA2* germline mutation are susceptible to breast cancer and other types of cancer. Using the immunopurification-mass spectrometry method, scientists found that BRCA2 and USP11 can form a specific complex. The presence of USP11 can prevent BRCA2 from being degraded, and the DNA repair process can proceed smoothly [13], which avoids DNA damage. Inhibitor of apoptosis proteins (IAPs) can inhibit cell apoptosis. Some scholars have found that cellular IAP2 (cIAP2) are stable in a variety of cancer cells, and its stability is regulated by USP11. In patients with colorectal cancer and melanoma, the overexpression of USP11 prevents cIAP2 from being degraded, which is related to the low survival rate of patients [18]. H2AX is an essential double-strand break (DSB) site-binding protein for DNA repair. It can provide a platform for the subsequent recruitment and amplification of DNA repair proteins and signal factors. It can be ubiquitinated by RNF8/RNF168 and can be deubiquitinated by USP11 [19]. Scientists discovered that eukaryotic initiation factor 4B (eIF4B) is a substrate of USP11 that can stabilize and enhance the activity of eIF4B. The fatty acid synthase (FASN) induced PI3K-S6 kinase signal phosphorylate USP11, which can enhance its interaction with eIF4B [20]. Ribonucleic acid export protein 1 (RAE1) has previously been shown to be involved in spindle assembly checkpoint (SAC) regulation and bipolar spindle formation. Recent studies have found that it is also a deubiquitinated substrate of USP11, which can explain USP11 indirectly participates in SAC regulation and bipolar spindle formation [21]. Recent studies have also found that USP11 can remove the K63-linked ubiquitin chain on E2F transcription factor 1 (E2F1), preventing E2F1 from degrading in the nucleus. When USP11 is downregulated, the stability and protein level of E2F1 decrease, which in turn reduces the mRNA level of *Peg10*^22^. It has recently been discovered that USP11 can compete with some E3 ubiquitinated proteases to regulate the stability of some proteins. For example, CRL4^DCAF8^ and USP11 counter-regulate the stability of myeloid leukemia factors (MLFs), and TRIM32/USP11 together balance the stability of ARID1A [22, 23]. Scientists detected an interaction between p53 and USP11 by coimmunoprecipitation and further found that USP11 and p53 can form a specific complex and stabilize p53 through deubiquitination [24]. There are still some proteins that interact with USP11 that are not described in this section, and they are mentioned in the relationship between USP11 and cancer. Collectively, these results demonstrated that USP11 plays an extremely important biological role by avoiding the degradation of some target proteins.

#### 2.2.2. Signaling Pathways Regulated by USP11

**Figure 2:**
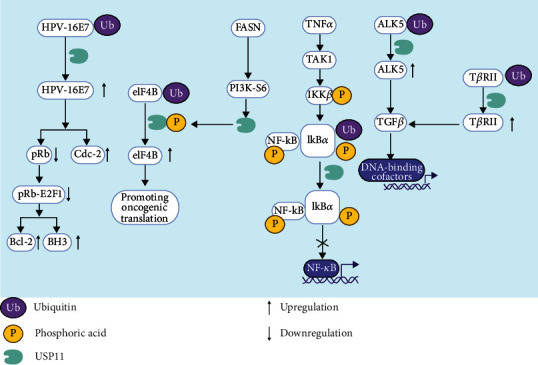
USP11 regulates signaling pathways through deubiquitinating interacting proteins. HPV-16E7: human papillomavirus-16E7 protein; pRb: retinoblastoma protein; Cdc-2: cell division cycle-2 protein; E2F1: E2F transcription factor 1; Bcl-2: B-cell lymphoma-2 protein; BH3: BH3-like motif-containing cell death inducer; FASN: fatty acid synthase; PI3K-S6: phosphoinositide 3-kinase regulatory subunit 6; eIF4B: eukaryotic initiation factor 4B; TNF*α*: tumor necrosis factor-*α*; TAK1: transforming growth factor kinase 1; IKK*β*: I*κ*B kinase *β*; NF-*κ*B: nuclear factor-kappa B; I*κ*B*α*: IkappaB *α*; ALK5: type I TGFb receptor; TGF*β*: transforming growth factor *β*; T*β*RII: TGF*β*-1 receptor II.

Many important functions of USP11 are achieved by interacting with proteins to regulate signal pathways. Therefore, we will detail the important signal pathways regulated by USP11. HPV-16E7 is a transforming protein related to the occurrence and development of cervical cancer. USP11 can combine with HPV-16E7 to form a specific complex to avoid degradation of HPV-16E7 and increase its stability. USP11 can regulate a variety of biological functions by stabilizing HPV-16E7 [25]. In one way, HPV-16E7 can degrade pRb, which is a typical tumor suppressor gene, so USP11 exhibits cancer-promoting property in the pathway. In addition, USP11 can also induce the upregulation of Bcl-2, BH3, and Cdc-2 by stabilizing HPV-16E7 [25]. It is clear that USP11 participates in the TNF-*α*-IKK*β*-I*κ*B*α*-NF-*κ*B signaling pathway. USP11 colocalizes with I*κ*B*α* and acts as an I*κ*B*α* deubiquitinase to inhibit the ubiquitination and degradation of I*κ*B*α* induced by TNF-*α*. Therefore, USP11 plays a key role in the downregulation of NF-*κ*B activation [26]. Additionally, USP11 is also involved in the TGF*β* signaling pathway. USP11 can resist the negative effect of SMAD7 on the TGF*β* signaling pathway. USP11 interacts with the type I TGF*β* receptor (ALK5) and causes its deubiquitination and stabilization, leading to TGF*β*-induced enhanced gene transcription [27]. Studies have used mitoxantrone (MTX), a USP11 inhibitor, to inhibit or downregulate USP11, resulting in increased T*β*RII ubiquitination and decreased T*β*RII stability. Subsequently, the TGF*β* signaling pathway was weakened, the phosphorylation level of SMAD2/3 decreased, and the expression level of fibronectin (FN) and smooth muscle actin (SMA) decreased [28]. These results further confirmed the role of USP11 in regulating multiple signaling pathways in cells. The signaling pathways regulated by USP11 are graphically depicted in Figure 2.

## 3. USP11 and Cancer

### 3.1. Regulating Cell Cycle and Proliferation

USP11 can regulate cell cycle and cell proliferation by interacting with some proteins. Microtubules constitute the nucleus, which is a very significant part in the process of cell division. RanBPM, a RanGTP-binding protein, is an indispensable protein in this process. USP11 can participate in the correct division of cells by deubiquitinating and stabilizing RanBPM [12]. The correct entry of chromatids into daughter cells during mitosis is a crucial step in maintaining the stability of the genome. In this process, RAE1 plays an important role as a protein involved in SAC regulation and bipolar spindle formation. Studies have found that USP11 regulates the deubiquitination of RAE1 on the mitotic spindle, which in turn regulates the formation of the spindle [21]. This can prevent the occurrence and development of cancer. The normal expression of USP11 plays a pivotal role in the normal division and proliferation of cells, but abnormal expression of USP11 can lead to tumors. For example, when USP11 is overexpressed, it can not only transform normal cells into cancer cells but also inhibit cell necrosis and apoptosis by stabilizing x-linked inhibitor of apoptosis protein (XIAP), thereby promoting the occurrence of tumors [29]. In addition, USP11 can also deubiquitinate and stabilize Mgl-1 in a RanBPM-dependent manner, thereby inhibiting the proliferation of cancer cells [30]. Therefore, the normal expression of USP11 is necessary for the correct division of cells.

### 3.2. DNA Repair

The correct repair of DNA damage is the process of maintaining the normal living state of cells, and no mistakes are allowed; otherwise, it will bring disastrous damage to cells. When the repair process fails, the apoptosis process will be initiated. If the apoptosis process cannot proceed smoothly, the cells will undergo malignant transformation. Compared with Lys^27^-, Lys^29^-, and Lys^48^-linked and linear chains, USP11 is more inclined to utilize Lys^63^-, Lys^6^-, Lys^33^-, and Lys^11^-linked chains to play a role in DNA repair [9]. As mentioned earlier, USP11 can participate in the DNA repair process by stabilizing BRCA2. In addition, another study found that homologous recombination (HR) proteins BRCA1 and BRCA2 cannot repair DNA damage when there is no USP11 existence [14]. The latest research also found that when MYCN is dephosphorylated at Thr58, USP11 can specifically bind to MYCN. Next USP11, BRCA1, and MYCN are mutually stable on chromatin [31]. There is a huge discovery that USP11 acts as a histone deubiquitinase to catalyze the deubiquitination of H2AK119 and H2BK120, terminate the reorganization of DNA and chromatin structure in time, and allow time for DNA repair. This shows that USP11 is a chromatin modifier and plays a key role in the DNA damage response and the maintenance of genome stability [32].

### 3.3. USP11 and Colorectal Cancer

The overexpression of USP11 is related to the poor prognosis of colorectal cancer. Studies have found that USP11 is overexpressed in colorectal cancer tissues. Whether *in vivo* or *in vitro* experiments, overexpression of USP11 can promote the growth and metastasis of colorectal cancer cells. Researchers found that USP11 stabilizes PPP1CA by deubiquitinating and protecting PPP1CA from proteasome-mediated degradation. Finally, the USP11/PPP1CA complex promotes the progression of colorectal cancer by activating the ERK/MAPK signaling pathway [33]. Another study also found that circ_DOCK1 inhibits the progression of colorectal cancer by targeting miR-132-3p to interfere with the expression of USP11 [34]. Taken together, these results suggest that USP11 is involved in the occurrence and development of colorectal cancer.

### 3.4. USP11 and Breast Cancer

The high expression of USP11 is also related to the poor prognosis of breast cancer. USP11 can enhance the epithelial-mesenchymal transition induced by TGF*β* and the continuous self-renewal of human breast epithelial cells. Upregulation of USP11 can increase the *in vitro* invasion and *in vivo* metastasis of breast cancer cells [35]. Most breast cancer patients express estrogen receptor *α* (ER*α*) and rely on it for proliferation and differentiation. In a cohort analysis of breast cancer patients, the high expression of USP11 was significantly correlated with the low survival rate of ER*α*-positive patients [36]. Therefore, the development of drugs targeting USP11 to treat breast cancer is imminent.

### 3.5. USP11 and Other Types of Cancer

USP11 is also involved in the occurrence and progression of other cancers. For example, USP11 regulates the stability of promyelocytic leukemia (PML) protein to inhibit the various malignant features of Notch-induced aggressive gliomas. The downregulation of USP11 and PML induced by Notch/Hey1 not only confers multiple malignant characteristics of aggressive gliomas, including proliferation, invasiveness, and cancer growth in orthotopic mouse model, but also enhances patient-derived glioma initiating cells the ability of self-renewal and tumor formation [37]. In gliomas, USP11 exhibits antitumor property. Zhang et al. found that USP11 can promote the migration and invasion of liver cancer cells in *vitro* and in *vivo* [39]. Wang et al. found that USP11 can also promote epithelial-mesenchymal transition of ovarian cancer by deubiquitinating Snail [38]. Therefore, USP11 is also a potential therapeutic target in ovarian cancer. Finally, USP11 can also control the stability of ARID1A by competing with TRIM32 to determine whether it is cancer-promoting or cancer-suppressing. Research has found that TRIM32 depletion can inhibit the proliferation, metastasis, and chemotherapy resistance of squamous cell carcinoma (SCC) by stabilizing ARID1A, while USP11 depletion can promote the development of SCC by promoting the degradation of ARID1A [23]. Our previous study found that USP11 can inhibit the proliferation, invasion, and metastasis of renal clear cell carcinoma by deubiquitinating vestigial-like4 (VGLL4). Therefore, USP11 can be used as a tumor suppressor in renal clear cell carcinoma [39].

Through the above review, we found that USP11 plays a dual role. USP11 plays different roles in different types of cancer, not only promoting cancer but also inhibiting cancer. The different roles of USP11 in different cancers can be intuitively found in Table 2.

### 3.6. USP11 as Prognostic Marker of Cancer

So far, USP11 has not been reported as a marker for diagnosing cancer, but it has been reported as a prognostic marker for cancer patients. On the one hand, it can be used as a prognostic factor after neoadjuvant therapy in breast cancer patients; low USP11 expression is independently associated with better survival prognosis [40]. On the other hand, it can be used as a marker of poor prognosis and metastasis in hepatocellular carcinoma and colorectal cancer [33, 41]. Therefore, the diagnostic and prognostic value of USP11 in cancer patient needs to be further developed.

## 4. USP11 and Targeted Therapy

Since USP11 plays an important role in the occurrence and development of a variety of cancers, it can be used as a potential therapeutic target to treat cancer. Researchers have screened 2000 compounds and found that mitoxantrone is an effective inhibitor of USP11, and mitoxantrone can significantly inhibit the activity of USP11 [42].

Mitoxantrone as an antitumor drug for clinical trials has a long history, in the treatment of a variety of tumors, and also received significant curative effect, such as breast cancer [43], ovarian cancer [44], lymphoma [45], and prostate cancer [46]. At present, mitoxantrone combined with other antitumor drugs is more commonly used to treat tumors, and its therapeutic effect is better than that of mitoxantrone alone. Low response rate and side effects are also prominent problems. Clinical trial targeting USP11 using mitoxantrone has not been reported.

Up to now, no other drugs targeting USP11 have been discovered. Therefore, the development of more effective targeted drugs for USP11 is an urgent problem to be solved.

## 5. Discussion

As a member of the deubiquitination protein family, USP11 performs biological functions mainly by deubiquitinating and stabilizing the target protein. After the target protein is ubiquitinated by the ubiquitinating enzyme, the target protein will then be labeled with ubiquitin and will be degraded immediately. However, due to the existence of USP11, it can disassemble the ubiquitin label on the target protein, allowing the target protein to continue to perform its biological functions.

USP11 participates in different biological functions by targeting different protein deubiquitination, so its biological behavior has a dual role, which depends entirely on the biological behavior of the target protein. The proteins that interact with USP11 and their downstream biological functions are listed in Table 3. When USP11 deubiquitinates and stabilizes oncoproteins, it plays the role of oncogene. For example, USP11 stabilizes eIF4B, which acts as a transcription factor to increase the mRNA translation of tumor proteins such as cMYC, BCL2, and BCL6, thereby promoting oncogenic translation [20]. When USP11 deubiquitinates and stabilizes tumor suppressor proteins, it plays the role of tumor suppressor gene. For example, USP11 can prevent the progression of squamous cell carcinoma by stabilizing ARID1A [23]. In addition to inhibiting cancer progression by stabilizing tumor suppressor proteins, USP11 can also inhibit the occurrence of tumors by stabilizing proteins involved in DNA repair. For example, USP11 participates in DNA repair by deubiquitinating P53, BRCA2, and H2AX. The smooth progress of the cell cycle is an important link to ensure that cells do not become cancerous. Simultaneously, USP11 also participates in stabilizing cell cycle-related proteins. For instance, the stability of RAE1 is a key factor in ensuring SAC regulation and bipolar spindle formation during mitosis [21]. Due to the different expression abundances of tumor-related proteins that can interact with USP11 in different cancer types, USP11 exhibits different oncogenic or tumor-suppressive roles in different cancer types. In addition, the expression levels of USP11 in different normal tissues are greatly different, and the expression levels in different cancers are also different, so the role of USP11 in tumors cannot be generalized. The biological function of USP11 should be implemented in specific cancer types.

In view of the dual role of USP11 in cancer, its dual effects need to be considered when designing drugs to target USP11. Inhibitors targeting USP11 should be designed in cancers promoted by USP11, and drugs that promote the expression of USP11 should be designed in cancers inhibited by USP11. So far, only mitoxantrone has been developed as a targeted inhibitor of USP11. Drugs that promote USP11 expression have not yet been developed.

Up till now, a large number of studies have focused on the downstream proteins regulated by USP11, and there are few studies on the upstream proteins of USP11, such as those that can regulate the transcription and translation of USP11 and enhance its activity. We believe that the upstream protein of USP11 has great research space and more important biological value. When the upstream protein of USP11 is well studied, we can regulate its expression by regulating its upstream, which will bring revolutionary changes to the treatment of cancers related to USP11.

Due to the dual role of USP11 in cancer, not only more specific targeted drugs for USP11 need to be developed, but also upstream proteins controlling USP11 expression need to be explored creatively. This will not only improve our comprehensive understanding of the biological behavior of USP11 but will bring light to the treatment of cancer patients.

## Figures and Tables

**Table 1:** Subtypes of USP11 and their biotype. The data from http://asia.ensembl.org.

Name	Transcript ID	bp	Protein	Translation ID	Biotype
USP11-204	ENST00000377107.7	3196	920aa	ENSP00000366311.2	Protein coding
USP11-201	ENST00000218348.7	3338	963aa	ENSP00000218348.3	Protein coding
USP11-202	ENST00000377078.2	1077	140aa	ENSP00000366279.2	Protein coding
USP11-203	ENST00000377080.7	804	49aa	ENSP00000366282.3	Protein coding
USP11-206	ENST00000469080.5	3467	No protein	-	Retained intron
USP11-207	ENST00000478596.5	970	No protein	-	Retained intron
USP11-209	ENST00000488848.1	904	No protein	-	Retained intron
USP11-212	ENST00000497179.1	900	No protein	-	Retained intron
USP11-208	ENST00000480104.5	697	No protein	-	Retained intron
USP11-205	ENST00000467378.1	696	No protein	-	Retained intron
USP11-211	ENST00000489111.1	516	No protein	-	Retained intron
USP11-210	ENST00000489030.1	340	No protein	-	Retained intron

**Table 2:** Different roles of USP11 in different cancers.

Cancer types	The function of USP11
Colorectal cancer	Promotes growth and metastasis [47]
Breast cancer	Increases invasion and metastasis [40]
Gliomas	Inhibits various malignant features [37]
Liver cancer	Promotes the migration and invasion [41]
Ovarian cancer	Promotes epithelial-mesenchymal transition [38]
Squamous cell carcinoma	Promotes proliferation and metastasis [23]
Renal clear cell carcinoma	Inhibits proliferation, invasion, and metastasis [39]

**Table 3:** The proteins are deubiquitinated and stabilized by USP11 and its downstream effects.

Proteins	Effect
RanBPM [12]	Microtubule formation
BRCA2 [13]	DNA repair
cIAP2 [18]	Promoting apoptosis
H2AX [19]	DNA repair
eIF4B [19]	Promoting oncogenic translation
RAE1 [21]	Bipolar spindle formation
E2F1 [48]	Driving *Peg10* gene expression
ARID1A [23]	Tumor suppressor function
MLFs [23]	Hematopoiesis and cancer
p53 [24]	Tumor suppressor function
Mgl-1 [31]	Inhibiting cancer
XIAP [29]	Promoting cancer
VGLL4 [39]	Tumor suppressor function
PPP1CA [33]	Activating ERK/MAPK signal
PML [38]	Tumor suppressor function
Snail [38]	Promoting EMT

Abbreviation: RanBPM: RanGTP-binding protein; BRCA2: breast cancer type 2 susceptibility protein; cIAP2: cellular inhibitor of apoptosis proteins; H2AX: histone H2AX; eIF4B: eukaryotic initiation factor 4B; RAE1: ribonucleic acid export protein 1; E2F1: E2F transcription factor 1; ARID1A: AT-rich interactive domain-containing protein 1A; MLFs: myeloid leukemia factors; p53: p53 protein; Mgl-1: Mgl-1 protein; XIAP: x-linked inhibitor of apoptosis protein; VGLL4: vestigial-like4; PPP1CA: serine/threonine-protein phosphatase PP1-alpha catalytic subunit; PML: promyelocytic leukemia; Snail: Zinc finger protein SNAI1; EMT: epithelial-mesenchymal transition.
